# Lymph Drainage of Posttraumatic Edema of Lower Limbs

**DOI:** 10.1155/2018/7236372

**Published:** 2018-03-05

**Authors:** Ana Carolina Pereira de Godoy, Rodrigo Ocampos Troitino, Maria de Fátima Guerreiro Godoy, José Maria Pereira de Godoy

**Affiliations:** ^1^Department of Santa Casa de São Paulo and Research Group of Clínica Godoy, São Jose do Rio Preto, SP, Brazil; ^2^ABC Medicine School, Santo André and Research Group of Clínica Godoy, São Jose do Rio Preto, SP, Brazil; ^3^Faculty of Medicine of São José do Rio Preto (FAMERP) and Research Group of Clínica Godoy, São Jose do Rio Preto, SP, Brazil; ^4^Cardiovascular Surgery Department, The Medicine School in São José do Rio Preto (FAMERP) and CNPq (National Council for Research and Development), São Jose do Rio Preto, SP, Brazil

## Abstract

**Objective:**

The present study was aimed at evaluating the use of mechanical and manual lymphatic therapy as a treatment for lymphedema resulting from orthopedic surgery that became painful after an episode of erysipelas.

**Case Report:**

A 70-year-old male patient suffered direct trauma resulting in a compound fracture of the tibia and fibula of the left leg. He was treated with an external fixator for four months followed by plaster cast immobilization for three weeks. He presented with fever and paresthesia in the lower left limb that resulted in an episode of erysipelas, and the patient evolved with painful lymphedema. Treatment using the Godoy and Godoy technique was proposed, including manual and mechanical lymphatic therapy. Water displacement volumetry was used to quantify the leg size reduction.

**Results:**

After 10 sessions of therapy, the patient presented a significant reduction in the limb volume and remission of symptoms.

**Conclusions:**

The method used may be a promising option for the treatment of posttraumatic edemas with pain.

## 1. Introduction

Traumatic injuries can affect all systems. Traumatic injuries of the musculoskeletal system are classified as direct or indirect, ranging from grazing, abrasions, lacerations, ruptures, crushing, and avulsions to complex tissue failure and fractures of various types. Etiological factors are diverse and include automobile accidents, sports accidents, explosions, burns, and injuries by firearms or sharp instruments, among others [1].

Mechanical injuries of soft or bony parts are usually followed by chronic edema, both at the site of the trauma and distally to it. This complication affects almost all patients with fractures of the lower limbs, whether they are submitted to surgery or not. The so-called “posttraumatic edema” may originate from lymphatic obstruction, deep venous thrombosis (DVT), or hyperactivity of growth factors and cytokines at the trauma site [2].

Erysipelas is an infectious cutaneous process caused by a bacterium that spreads through lymphatic vessels. The port of entry may be a skin injury due to trauma, ulcers, or, more commonly, interdigital mycosis, popularly known as “athlete's foot” [3]. The affected area presents erythema, edema, hyperthermia, and pain, and as systemic symptomatology, the patient may present with chills, high fever, asthenia, headache, nausea, and vomiting. Erysipelas is considered an important complication in both edema and trauma cases [3, 4].

Edema of the limb favors erysipelas infections due to insufficient venous and lymphatic circulation, while erysipelas becomes an aggravating factor for lymphedema as a consequence of relapsing outbreaks [5].

One of the ways of treating posttraumatic lymphedema is lymph drainage, and in recent years, new techniques of manual and mechanical lymph drainage have been developed by Godoy and Godoy [6, 7].

In relation to the technique, Godoy and Godoy developed a new technique to stimulate the lymphatic system which was recently named manual lymphatic therapy (MLT), the therapy using linear movements which manually displace the lymph along the anatomic path of the lymphatic vessels. The Godoy and Godoy technique was developed based on the normal anatomy and on physiology and pathophysiological processes and adapted for each type of lymphedema. MLT obeys the concepts of the hydrodynamic principles needed to drain collectors [6]. Mechanical lymph drainage has the potential to drain both the superficial and deep lymphatic chains and uses an electromechanical device, RAGodoy®, that performs continuous passive flexion and extension of the ankle [8, 9].

The present study was aimed at evaluating the use of mechanical and manual lymphatic therapy as a treatment for lymphedema due to orthopedic surgery that became painful after an episode of erysipelas.

## 2. Case Report

A 70-year-old male patient suffered direct trauma resulting in a compound fracture of the tibia and fibula of the left leg. He was treated with an external fixator for four months followed by plaster cast immobilization for three weeks. After removal of the cast, he presented edema and was referred for physiotherapy where he performed five sessions of manual lymph drainage and six sessions of hydrotherapy without resolving the condition. The patient presented with fever and paresthesia of the left leg. A physical examination identified interdigital mycosis between the toes of the left foot, and the medical diagnosis was erysipelas.

After treating erysipelas, the patient presented hypersensitivity, pain, and worsening of the edema. The patient was referred for clinical treatment. The method proposed included manual lymph drainage (Godoy and Godoy technique) [6, 7], the technique using linear movements which manually displace the lymph along the anatomic path of the lymphatic vessels. The Godoy and Godoy technique was developed based on the normal anatomy and on physiology and pathophysiological processes and adapted for each type of lymphedema and obeys the concepts of the hydrodynamic principles needed to drain collectors, and a mechanical lymph drainage (RAGodoy device), an apparatus that performs plantar flexion and dorsiflexion exercises, was utilized to dynamically evaluate venous pressure variations during passive exercising, for two hours per day for ten consecutive days [8, 9]. Water displacement volumetry was used to quantify the leg size reduction. Before starting treatment, the difference between the edematous leg and the contralateral limb was 567 mL.

**Figure 1:**
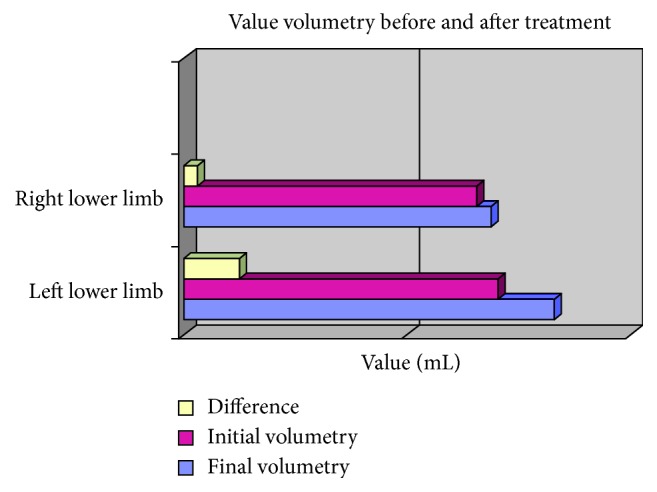
Leg volumes before and after treatment.

After the first session, the patient had less hypersensitivity with the pain improving. Treatment reduced the affected leg by about 497 mL, that is, 87% of the excess volume. Moreover, there was a reduction of 129 mL of the normal leg volume. The initial (pretreatment) and final (after ten treatment sessions) volumes of both legs are shown in Table 1 and Figure 1. This study was approved by the Research Ethics Committee of FAMERP (#20445-11/05/2012).

## 3. Discussion

The present study reports the case of a patient who suffered a compound fracture, with edema and erysipelas developing as complicating factors. The choice of treatment was manual lymph drainage (Godoy and Godoy method) [6, 7] and mechanical lymph drainage (RAGodoy), a method developed for the treatment of edema due to impairment of the lymphatic system [8, 9].

Mechanical lymph drainage was chosen because there is involvement of both the superficial and deep lymphatic systems after trauma, and the RAGodoy equipment performs lymph drainage of both systems [8, 9].

Studies have shown that 10.5% of patients with edema due to traumatic injuries presented lymphatic lesions confirmed by lymphoscintigraphy examination, 23.6% presented edema as a consequence of deep venous thrombosis, and 65.9% of patients presented edema related to inflammations. The increase in lymphatic flow in the latter cases can be explained by changes in capillary permeability due to the inflammatory process [2].

The use of RAGodoy during postsurgical hospitalization may be useful as an auxiliary therapy to prevent cases of DVT, as it passively performs flexion and extension movements of the ankle, promoting the activation of the calf muscle pump [8, 9]. The use of this device when DVT already exists is contraindicated.

Erysipelas, pain, and increased sensitivity are not exclusion criteria for the use of the equipment to treat lymphedema; in the current case, the patient benefited from improved pain and decreased hypersensitivity.

The association of mechanical lymphatic therapy with manual lymphatic therapy is important as manual lymph drainage can help reduce edema at the trauma site more specifically than mechanical lymph drainage.

## 4. Conclusion

Mechanical lymph drainage (RAGodoy) associated with manual lymph drainage was effective to reduce edema and pain in lymphedema resulting from a traumatic injury and aggravated by erysipelas.

## Figures and Tables

**Table 1:** Volumetric values (in mL) at the start (pretreatment) and end of treatment (after 2 hours daily for 10 days).

	Initial volume (mL)	Final volume (mL)	Difference (mL)
Left leg	3316	2819	497
Right leg	2749	2626	123
